# Assessment of contamination of natural waters with radionuclides and heavy metals the case of Karabulak creek at the Semipalatinsk Test Site

**DOI:** 10.1371/journal.pone.0310833

**Published:** 2025-02-06

**Authors:** Madina Dyussembayeva, Almira Aidarkhanova, Azhar Tashekova, Yerbol Shakenov, Vladimir Kolbin, Alyona Merkel, Fail Zhamaldinov, Natalya Larionova, Igor Gorlachev, Pavel Kharkin, Sergey Lukashenko, Yelena Yevlampiyeva

**Affiliations:** 1 Department of Monitoring and Ecological Analytical Research, Institute of Radiation Safety and Ecology, National Nuclear Center RK, Kurchatov, Kazakhstan; 2 Laboratory of Low-Background Measurements of the Center for Integrated Environmental Research, Institute of Nuclear Physics, Almaty, Kazakhstan; 3 Center for Integrated Environmental Research, Institute of Nuclear Physics, Almaty, Kazakhstan; 4 Laboratory of Radiochemistry and Analytical Chemistry, Russian Institute of Radiology and Agroecology, Obninsk, Russia; 5 Scientific Activities Management Department, Shakarim University, Semey, Kazakhstan; King Abdulaziz University, SAUDI ARABIA

## Abstract

This paper presents research data on water contamination levels of artificial radionuclides and heavy metals in Karabulak Creek at the Semipalatinsk Test Site. The uranium isotopic composition has been studied (^235^U and ^238^U). Priority contaminants and the most ‘contaminated’ sections of the Karabulak creek were identified, and water quality-related indicators were also estimated. The environmental situation around Karabulak Creek at the «Degelen» site—at which underground nuclear tests have been conducted—was found to be unique and attributed to the combined effect of man-made radiation and natural factors and, in particular, to geochemical features of the region.

## 1. Introduction

The Semipalatinsk test site (STS) was one of the world’s largest nuclear weapon proving grounds, occupying 18,311.4 km^2^. During its operating period (1949–1989), 456 nuclear tests were conducted, of which 30 were aboveground, 86 were in the air, and 340 were underground.

Nuclear blasts were conducted only at dedicated sites, rather than throughout the territory. The content of different radionuclides released into the environment during atmospheric tests was in the order of 17.6 PBq (0.476 MCi) [1, 2]. The problem of nuclear effects on the environment and the public living in close proximity to test locations remains a challenge in radiobiology.

Conventionally, the STS is mostly known as a source of radiation hazard to the local public. However, the ecological situation around this site may also be highly adverse owing to the impact of heavy metals, as suggested by available experimental data [3–8] and that there are various mineral deposits within the test site.

The STS has large mineral deposits, such as gold in «Naimanzhal», «Baitemir», and «Koskuduk»; coal in «Karazhyra»; and fluorite in «Karadzhal» [9, 10]. Currently, unauthorised agricultural activities, such as year-round sheep and horse grazing, are ongoing at this site. Additionally, this area is actively used for industrial activities, such as the development of deposits and laying of power lines.

Most research on terrestrial [11–13] and aquatic [14–20] ecosystems in the STS is currently dedicated to the behaviour of artificial radionuclides. High concentrations of these and heavy metals may reach considerable quantities in surface STS water bodies, posing a potential hazard to ecosystems and local health.

At the «Degelen» underground nuclear blast site, the Karabulak Creek intersecting the «Karadzhal» deposit is a useful site to study the redistribution of radioactive elements and heavy metals in the ecosystem. The water of the Karabulak Creek is not potable; however, farm cattle graze, including on hay, and drink ubiquitously in the Karabulak Creek valley. This may lead to the accumulation of heavy metals and radionuclides in humans through the water–animal–human food chain.

Previous research has shown that the major radionuclides contaminating Karabulak Creek are ^3^H [13, 14, 18], ^90^Sr [15, 16, 21], and ^137^Cs [21]. The ^3^H content in the surface water of Karabulak Creek reached 30 kBq/kg [21], and in the ground water (occurrence depths of 0.7 to 2 km) of Karabulak Valley, they ranged from <7 to 2000 Bq/kg [22].

This study aimed to assess the ecological conditions of STS natural waters in Karabulak Creek and to investigate the combined effect of artificial radionuclides and heavy metal contamination.

## 2. Materials and methods

### 2.1 Study area

The test site of underground nuclear blasts at «Degelen» is located in the Degelen mountain range, an isometric granite massif of ∼220 km^2^. The dominant landform type in the study area is low hummocks 300–500 m high, dominated by dome- and cone-shaped hills. The highest mountain peak is just above 1000 m [23].

From 1961 to 1989, as per different estimates, the number of blasts was roughly 210, and 181 mine workings (tunnels) were driven at the «Degelen» site [1]. Due to underground nuclear blasts, the rocks became more permeable owing to the deformation of the rock mass, producing extensive cavities, fracturing zones, and spaces in the tunnels themselves. Radionuclide-contaminated water, while moving through crack systems and the tunnel cavity, recharge ground water basins or flow out onto surfaces in the vicinity of a tunnel entry [14]. Water streams from most tunnels within the creek valleys of the «Degelen» site are generally dozens or hundreds of metres long. Radioactive contamination was found, not only in tunnel cavities but also on the surface, particularly in natural ecosystems of creek valleys at the «Degelen» site that were most exposed to this contamination [14, 16, 24].

**Fig 1:**
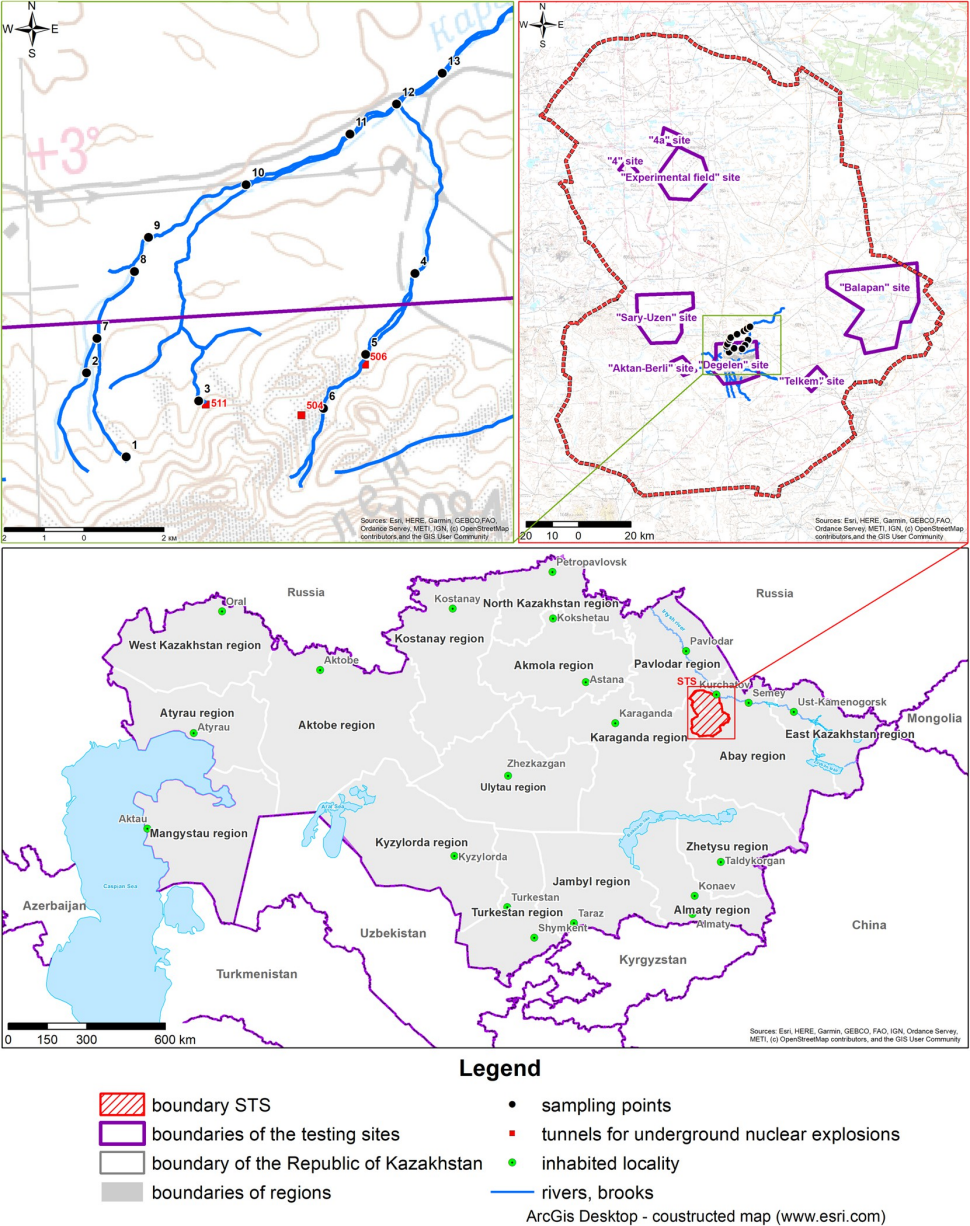
Schematic map of the STS location and sampling points at Karabulak Creek [13]. Republished from https://creativecommons.org/licenses/by/4.0/2024 under a CC BY 4.0., with permission from Abisheva Mariya, original copyright 2024.

Karabulak Creek originates from the northern Degelen Mountain range and is one of the largest water streams (Fig 1). The creek bed is slightly pronounced and traceable up to 40 km away from the mountain range. Several tributaries exist within the «Degelen» site, and the creek valley extends northeast. Karabulak Creek is genetically linked to the watercourses of adits No. 504, 506, and 511, which have steady water seepage and are the major sources of radionuclide creek contamination (Fig 1).

**Fig 2:**
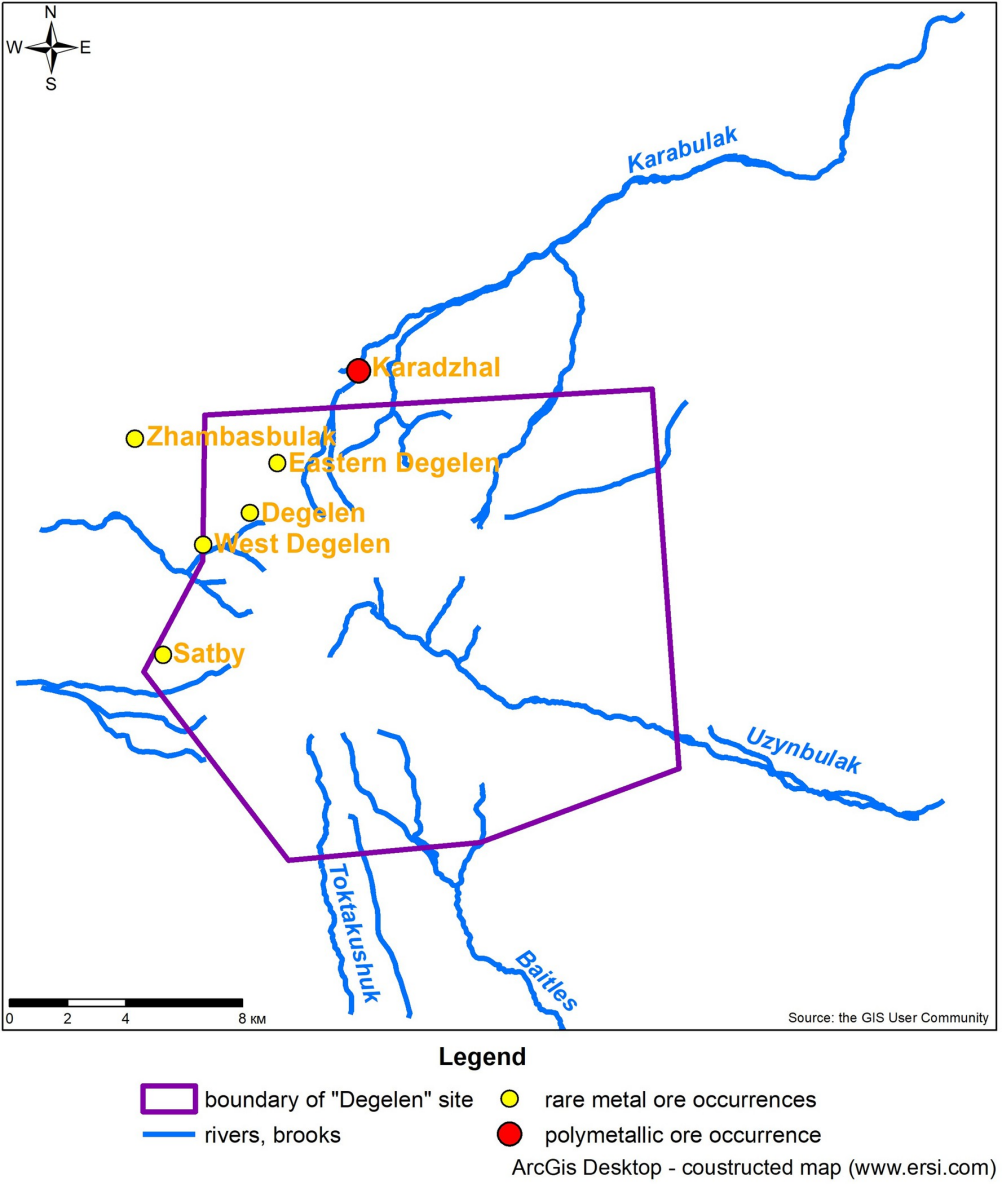
Location map of the Karabulak creek in the Degelen mountain range showing the mineral deposits [18]. Republished from https://creativecommons.org/licenses/by/4.0/2024 under a CC BY 4.0., with permission from Abisheva Mariya, original copyright 2024.

The Karabulak catchment contains five ore occurrences (Fig 2) capable of influencing the trace element composition of the creek water. Four of the five ore occurrences are mineralised in rare metals.

The Degelen deposit is a typical rare metal site. Rare metal salinity is spatially and genetically linked to leucocratic and alaskite granites. The ores contain wolframite, haematite, pyrite, sphalerite, galena, scheelite, molybdenite, beryl, and other minerals. The most common chemical elements in this region are tungsten, beryllium, and molybdenum. Zirconium, antimony, and tin also occur. Ore occurrences are in Eastern and Western Degelen and Zhambasbulak, and zirconium occurs in Satbai on the deposit flanks. The study area occurs in a uranium-bearing province and some local areas, such as the valley of Karabulak, have uranium anomalies [9].

Karabulak crosses the fluorite deposit «Karadzhal» (Fig 2), at which 9 ore skarn bodies were discovered. The largest body size was 500 × 100 m. Skarns of the following compositions occur: magnetite, garnet, garnet-vesuvian, pyroxene-garnet, and garnet-fluorite [25].

The bulk of the fluorite is in the quartz-fluorite vein, extending for 878 m. The ferrum content in magnetite skarns is 6.4–37%. The associated metals are copper (0.13–3%), lead (up to 1.2%) and zinc (0.19–1.14%).

The salinity of beryllium was mostly represented by chrysoberyl- and beryllium-containing vesuvian. The content of beryllium oxide, tungsten oxide, molybdenum, and tin was 0.2, 0.4, 0.02, and 0.02%, respectively [8, 22]. Thus, the development of the «Karadzhal» deposit can be an anthropogenic source of elevated concentrations of such elements as ferrum, beryllium, copper, lead, zinc, and molybdenum in the water.

### 2.2 Sampling

Water samples were collected during low water conditions in the summer of July 2018 because, in the spring and fall seasons, flood contaminants are greatly diluted and washed off from vast catchment areas. Figure (Fig 1) shows a schematic arrangement of the sampling points along the Karabulak Creek.

Water samples were collected and preserved as per GOST 31861–2012 [26], which applies to any water type and establishes general requirements for water sampling, transportation, and preparation to determine water composition indicators and properties.

When sampling water for element analysis, the following was performed: water filtration to remove mechanical impurities through a paper filter «blue ribbon»; samples preservation by adding the concentrated nitric acid (HNO_3_) of «very-high-purity» grade at the rate 3 ml of HNO_3_ per 1 L of water sample. The filtration and preservation were performed in situ. For general chemical water analysis (chlorides, sulphates, total hardness, and total salinity), 1.5 L water samples were collected without nitric acid preservation. All samples were stored at 4°C until further analysis.

### 2.3 Water sample analysis for chemical elements

The chemical element contents were determined by inductively coupled plasma mass spectrometry (ICP-MS) using an Agilent 7700x-type quadrupole mass spectrometer by Agilent Technologies (USA) and an «iCAP 6300 Duo» inductively coupled plasma atomic emission spectrometer (ICP-AES) by Thermo Scientific (USA). These techniques allowed the determination of the content of 20 elements: Li, Be, Al, Cr, Mn, Fe, Co, Ni, Cu, Zn, Bi, Nb, Mo, Cd, Cs, Ba, Sm, Eu, Pb, U with detection limits of 0.01 to 100 μg/L with a 10 to 15% uncertainty. It should be noted that the macro elements, Al and Fe, were predominantly determined by ICP-AES.

Spectrometer calibration uses 10 and 20 μg/L calibrating solutions for ICP-MS and 1000 and 5000 μg/L for ICP-AES. Multi-element reference standard solutions containing metals made by Perkin Elmer (USA) were used for calibration, with a rated certified value of metal content equal to 10 mg/L and a 0.5% certified uncertainty value (dilution factor k = 2).

The uranium isotope concentrations (^235^U and ^238^U) were determined using ICP-MS with a quadruple mass spectrometer (Agilent 7700, Agilent Technologies). SSS 7115–94-based solutions containing uranium ions (300 mg/L) were used to calibrate the mass spectrometer. Calibration was carried out at three points and the reference uranium solution concentrations were 30, 150 and 300 μg/L.

The measurement quality was verified by measuring the calibrating solution every 10 samples. To prepare accuracy control samples (validation) for calibration characteristics, RS Inorganic Ventures IV-ICP-MS-71A, CMS-1 (Inorganic Ventures, USA) containing metals were used with a rated certified value of metal content equal to 10 mg/L^-1^ and uncertainty of 0.5% (dilution factor k = 2). If the calibration result was unsatisfactory (deviation of the calibration graph by 8–10%), the instrument was recalibrated considering new background parameters.

The analysis was performed according to the ISO 17294-2-2019 procedure using ICP-MS [27]. To determine the chemical-physical parameters and the macro component content in water, a traditional wet chemistry analysis was carried out using standard methods [28]: pH level, salinity, hardness, and major ions of the basic composition (Na^+^, K^+^, Ca^2+^, Mg^2+^, Cl^-^, HCO^-3^, and SO_4_^2-^).

### 2.4 Water sample analysis for artificial radionuclides

#### 2.4.1 Determination of ^3^H content

The content of ^3^H in 0.02 L water samples was determined with a liquid scintillation spectrometer (TRI-CARB 2900 TR, Perkin Elmer, USA) as per ISO 9698:2019 [29]. A 5 mL subsample was collected from a filtered sample and placed in a 20 mL plastic vial. The ratio «sample–scintillator» (scintillation cocktail Ultima Gold LLT) was 1:3. The sensitivity of the measurement technique in use while determining ^3^H activity concentration in the free water was 12 Bq/kg.

#### 2.4.2. Determination of ^137^Cs, ^90^Sr, and ^239+240^Pu content

Samples were prepared and the activity concentrations of ^137^Cs, ^90^Sr, and ^239+240^Pu were determined according to the determination procedure for artificial ^137^Cs, ^90^Sr, and ^239+240^Pu in natural waters using the preconcentration technique. To control the chemical yield, a pre-filtered sample was spiked with isotopic labels of ^134^Cs, ^85^Sr, and ^242^Pu.

Water samples (10 L) were enriched by co-precipitation: ^241^Am and ^239+240^Pu with ferrum hydroxide (III), ^90^Sr with calcium carbonate, ^137^Cs with copper hexacyanoferrate. Next, by means of γ-spectrometric measurements, the content of ^137^Cs and ^241^Am was determined with a γ-spectrometer (BE 3830, Mirion, USA). For the spectrometer energy calibration, a set of standard γ-sources (OSGI) was used; for geometry calibration, volumetric measures of special-purpose specific activity («OMACH» Rosatom) were used containing the following radionuclides: ^137^Cs, ^152^Eu, and ^241^Am.

The ^90^Sr content was determined by β-spectrometry from ^90^Y following a radiochemical pre-isolation followed by measuring with a β-spectrometer (TRI-CARB 2900 TR, Perkin Elmer, USA) [30]. The accuracy of ^90^Sr activity determination was verified by periodic sample measurements prepared from a ^90^Sr–^90^Y solution of a known activity (in the bottle N^o^ 3623 «V.G. Khlopin Radium Institute»).

The content of ^239+240^Pu was determined by α-spectrometry following extraction-chromatographic isolation and by precipitation onto a metal disk followed by measuring with a α-spectrometer (Alpha Analyst, Mirion, USA) [31]. It should be noted that detection limits of analytical techniques in use are well below intervention level values (IL) for radionuclides of interest in potable water: ^137^Cs—11 Bq/kg, ^90^Sr—4.9 Bq/kg, ^239+240^Pu—0.55 Bq/kg, and ^3^H—7600 Bq/kg [32].

### 2.5 Quality control

Water samples were analysed in accredited laboratories (ISO 17025:2009) of the «Institute of Radiation Safety and Ecology» of the Republican State Enterprise on the REM «National Nuclear Centre of the Republic of Kazakhstan», Kurchatov. Research was undertaken using the analytical and test equipment calibrated and tested as per the Law of the Republic of Kazakhstan dated 7 June 2000 No. 53-II «On assurance of the uniformity of measurements».

The measurement quality while analysing the samples was monitored as follows: blank samples were prepared by accomplishing all stages without adding a sample. Laboratory control samples used to control the analytical process underwent all preparation stages as per the respective analytical procedure.

### 2.6 Processing of results

Maps quoted in this study were constructed by means of ArcGIS 10.8 software using digitised maps of the Republic of Kazakhstan that were acquired by the branch «Institute of Radiation Safety and Ecology» of NNC RK from the Republican State Enterprise «National Mapping and Geodesic Fund» of the Committee for Geodesy and Mapping, the Ministry of Digital Development, Innovations and Aerospace Industry of the Republic of Kazakhstan under the State Procurement Contract No. 02-19/122 dated 04/28/2020.

To assess water quality of the Karabulak Creek, the water complex contamination index (WCCI HM) for a group of heavy metals was applied given a hazard class [33]. The complex contamination index was computed separately for each hazard class, including all ingredients in the computational series that exceed their own maximum permissible concentration (MPC), that is:

$$WCCIHM_{j}=(\frac{1}{n})\cdot \sum_{i=1}^{n}\frac{C_{i}}{MPC_{i}},$$

where WCCI HM j is the index of water contamination with heavy metals of hazard class j, Ci the concentration of the i^th^ component of hazard class j (μg/L), MPCi the maximum permissible concentration of the i^th^ component of hazard class j (μg/L,), and n the number of components of hazard class j [33]. When classifying the water quality levels, the following contamination level sequences were used for water bodies: a) regulatory clean, b) moderately contaminated, c) heavily contaminated, and d) extremely contaminated (Table 1).

**Table 1:** Classification of water quality levels depending on heavy metal contamination.

Contamination level	Estimated figures of water body contamination from WCCI HM
Regulatory clean	≤2.0
Moderately contaminated	2.1–6.0
Heavily contaminated	6.1–10.0
Extremely contaminated	≥10.1

## 3. Results and discussion

### 3.1 Results

#### 3.1.1 Macro component composition of water in Karabulak Creek

For a convenient analysis of findings, sampling points were conventionally combined by territory into four tributaries and the main bed (Fig 1). The findings of the general chemical analysis of the water samples from Karabulak Creek are listed in Table 2.

**Table 2:** Chemical composition of Karabulak Creek.

Sampling point	Hardness (mmole/L)	pH	Cation content (mg/L)			Anion content (mg/L)			Salinity (mg/L)
			Na^+^+K^+^	Ca^2+^	Mg^2+^	Cl^-^	HCO^3-^	SO_4_^2-^	
Tributary 1									
T-1	3.4±0.1	7.7±0.2	23±3	48±3	11.0±2.0	9.0±1.0	122±3	100±6	243±15
Tributary 2									
T-2	1.7±0.1	7.2±0.2	53±10	30±7	2.0±0.1	10.0±2.0	202±10	21±1	198±4
Tributary 3									
T-3	7.3±0.4	7.3±0.2	34±6	97±6	30.0±1.0	10±1	366±1	116±6	446±6
Tributary 4									
T-4	9.7±0.3	7.7±0.1	75±2	133±12	140±5	56±16	267±104	232±31	735±148
T-5	7.5±0.1	7.6±0.1	30±15	95±9	33±6	16±8	351±2	125±22	473±53
T-6	6.0±0.5	7.5±0.1	4.7±1,2	93±12	16.0±3.5	38±16	100±5	167±38	352±23
Bed									
T-7	5.5±0.1	8.3±0.1	28±5	90±14	6.0±1.0	10.0±1.0	278±39	68±4	334±47
T-8	8.1±0.1	8.4±0.1	41±3	100±10	38.0±1.0	10.0±0.2	302±3	225±1	571±2
T-9	7.5±0.2	8.4±0.3	45±11	93±12	34.0±8.0	12.0±3.0	303±3	199±22	561±17
T-10	15.0±0.7	8.1±0.1	1886±873	120±26	110.0±16.0	290±10	975±443	2800± 1200	5300±2300
T-11	7.6±0.1	8.6±0.1	839±30	95±7	35.0±7.0	128±4	625±7	1450±70	2786±139
T-12	8.0±0.1	8.5±0.1	413±5	90±14	44.0±9.0	115±7	400±71	775±35	1674±23
T-13	11±2	7.2±0.1	311±20	110±14	68.0±10.0	110±14	450±71	675±35	1548±31
Mean±SD	**7.6±3.3**	**7.9±0.5**	**291±536**	**92±27**	**44±41**	**63±82**	**365±229**	**535±790**	**1171±1448**
MPC Kazakhstan [34]	**7**	**6–9**	**-**	**-**	**-**	**350**	**-**	**500**	**1000**

Note: SD: standard deviation.

The pH value of Karabulak water ranges widely from 7.2 to 8.6, and in most cases, the reaction is slightly alkaline. Water hardness indices of Karabulak Creek vary from «soft» to «very hard» (1.7 to 15 mmole/L). By salinity levels, the head water of Karabulak (T-1 to T-4) is fresh (0.2–0.7 g/L), predominantly containing hydro carbonates and calcium. Next, the total salinity of water downstream gradually rises, and in the lower reaches of Karabulak (T-10 to T-13) The water has a sulphate-sodium composition (1.5–2.8 g/L), referred to as saltish waters. The water in these sections did not conform to the Health Standards established by the Republic of Kazakhstan [34] for the content of sulphate-ions, hardness, and salinity indices.

One factor that forms the chemical composition of The waters of Karabulak Creek is related to the evaporative concentration, as indicated by the variation in the ratio of the main ions as the salinity increases. At points of elevated salinity (T-10 to T-13), sulphate ions become more dominant, and sodium significantly increases, which points to the geochemical evaporation barrier specific to surface water bodies in arid zones. The highest salinity was recorded at T-10 (saline waters: 5.3 g/L).

#### 3.1.2 Trace element composition of water in Karabulak Creek

The results of the mass spectrometric analysis of the water samples are listed in Table 3. The content of the following elements in each water sample tested does not exceed the detection limits: Nb < 0.01 (MPC = 10 μg/L), Cs < 0.01 (MPC = 0.5 μg/L), Sm < 0.01 (MPC = 24 μg/L), Eu < 0.01 (MPC = 300 μg/L), Bi < 0.1 (MPC = 100 μg/L).

**Table 3:** Trace element contents in water samples of Karabulak Creek (μg/L).

Sampling point	Li	Be	Al	Cr	Mn	Fe	Co	Ni	Cu	Zn	Mo	Cd	Ba	Pb	U
Tributary 1															
T-1	35±4	0.60±0.08	2000±200	69±5	63±7	390±40	0.98±0.02	21±5	1.5±0.1	140±20	280±30	<0.1	37±2	2,4±0,1	79±8
Tributary 2															
T-2	13±2	0.77±0.10	510±80	1.1±0.1	120±10	540±50	0.70±0.08	3.7±0.4	2.4±0.3	<0.005	8±1	<0.1	110±13	2,5±0,3	0.7±0,1
Tributary 3															
T-3	92±9	1.5±0.2	710±70	34±4	32±	200±20	0.62±0.07	5.7±0.7	<0,2	35±4	28±4	<0.1	22±3	<0,01	350±40
Tributary 4															
T-4	30±3	<0.02	180±20	13±2	83±10	130±14	1.0±0.1	6.0±0.7	0.8±0.1	25±3	<0.05	<0.1	56±7	<0,01	3.5±0.4
T-5	15±2	0.30±0.06	<5	1.9±0.2	80±9	50±5	0.41±0.05	<1	1.4±0.2	37±4	16±2	<0.1	31±4	<0,01	12±1
T-6	11±1	0.50±0.07	<5	1.3±0.2	97±10	60±7	0.42±0.05	8.4±1.0	13±2	100±12	38±5	1,4±0.3	34±3	43±4	6.2±0.7
Bed															
T-7	18±2	<0.02	620±60	25±10	160±20	290±35	0.96±0.01	5.6±0.7	1.3±0.1	100±12	15±2	<0.1	85±10	0,46±0,05	2.6±0.3
T-8	28±3	0.40±0.06	<5	<0.5	2.0±0.3	20±2	0.43±0.05	<1	1.0±0.1	67±8	57±7	<0.1	5.0±0.6	<0,01	24±2
T-9	28±3	<0.02	<5	<0.5	3.0±0.4	30±4	0.46±0.04	<1	0.5±0.2	43±5	55±6	<0.1	8.0±0.9	4,5±0,5	23±2
T-10	210±20	<0.02	<5	1.8±0.2	61±7	40±5	0.39±0.05	<1	13±2	37±4	1000±100	1,9±0,6	21±3	<0,01	140±10
T-11	140±10	<0,02	<5	<0.5	1100±100	3900±400	0.85±0.10	1.7±0.3	3.6±0.4	71±8	30±3	<0.1	41±10	<0,01	78±8
T-12	68±7	<0,02	<5	1.3±0.1	63±8	180±21	0.48±0,06	1.9±0.2	1.7±0.2	26±3	38±4	<0.1	23±3	<0,01	6.0±0.7
T-13	52±5	<0.02	<5	0.60±0.07	110±10	90±9	0.49±0.05	9.7±1.2	9.6±2.0	38±5	51±6	<0.1	22±2	13±2	47±5
**Mean±SD**	57±59	0.32±0,44	312±571	12±20	152±288	455±1047	0.63±0.24	5.2±0,5	3.8±4.7	55±39	124±273	0,34±0,60	38±30	5,1±12	59±97
**Hazard class**	**2**	**1**	**2**	**3**	**3**	**3**	**2**	**3**	**3**	**3**	**2**	**2**	**2**	**2**	**1**
**Average content in global river waters [36]**	**1.84**	**0.0089**	**-**	**0.7**	**34**	**66**	**0.15**	**0.80**	**1.48**	**0.60**	**0.42**	**0.08**	**23**	**0.08**	**0.37**
**MPC in Kazakhstan [34]**	**30**	**0.2**	**500**	**500**	**100**	**300**	**100**	**100**	**1000**	**5000**	**250**	**1**	**100**	**30**	**-**
**WHO’s standard [35]**	**-**	**-**	**-**	**50**	**80**	**-**	**-**	**70**	**2000**	**-**	**70**	**3**	**1300**	**10**	**30**

Excess MPC, according to the Health Standards of the Republic of Kazakhstan [34] in the water of Karabulak Creek, was found both in the Tributaries and in the main bed for the following elements: Be (1.5–7.5 Times), Li (1–7 Times), Fe (1.3–13 Times), Mn (1.1–11 Times), Mo (1.1–4 Times), Cd (1.1–2 Times), and U (1.6–12 Times). Tributaries 1, 2, and 3 had significant concentrations of Al in The water exceeding MPC by a factor of 1 to 4, and in Tributary 4 (T-6) it was 1.4 Times The lead content.

Karabulak Creek waters were contaminated by uranium exceeding regulatory values established by The World Health Organization [35] by 1.6 to 12 Times (MPC = 30 μg/L). Thus, MPC values exceeded 10 of The 20 chemical elements studied in The water of Karabulak Creek.

#### 3.1.3 Radionuclide composition of Karabulak water

In each water sample Tested, concentrations of most artificial radionuclides of interest did not exceed detection limits: ^241^Am < 0.17 Bq/kg (IL = 0.69 Bq/kg), ^239+240^Pu < 0.35 mBq/kg (IL = 550 mBq/kg), ^137^Cs < 0.37 Bq/kg (IL = 11 Bq/kg), ^152^Eu < 0.24 Bq/kg (IL = 98 Bq/kg), ^154^Eu < 1.3 Bq/kg (IL = 69 Bq/kg), ^155^Eu < 0.35 Bq/kg (IL = 430 Bq/kg). The exceptions were ^3^H and ^90^Sr (see Table 4 for their contents). ^3^H activity concentration in the test water samples ranged from 1600 to 76000 Bq/kg. The permissible intervention level for ^3^H, recorded in The water of Tributary 1 (T-1); Tributary 3 (T-3), Tributary 4 (T-4 to T-6) and the main bed (T-7 to T-9), was exceeded by 5.7 to 10 Times.

**Table 4:** ^3^H and ^90^Sr content in water samples from Karabulak Creek.

Sampling point	Sr-90 (Bq/kg)	H-3 (Bq/kg)
Tributary 1		
**T-1**	16±1	44000±4000
Tributary 2		
**T-2**	0.03±0.02	6200±600
Tributary 3		
**T-3**	130±10	76000±8000
Tributary 4		
**T-4**	18±2	22000±2000
**T-5**	26±3	25000±3000
**T-6**	1.2±0.1	18000±2000
Bed		
**T-7**	0.15±0.024	8200±800
**T-8**	<0.024	19000±2000
**T-9**	<0.022	19000±2000
**T-10**	0.2±0.02	1600±200
**T-11**	0.2±0.02	2800±300
**T-12**	<0.024	2200±200
**T-13**	0.069±0.024	1600±200
Intervention level [32]	4.9	7600

Concentrations of ^90^Sr exceeded IL (IL = 4.9 Bq/kg) in the water of Tributary 1 (T-1) by 3 Times, Tributary 3 (T-3) by 26 Times, and Tributary 4 (T-4, T-5) by 4 and 5 Times. respectively. Maxima of ^3^H and ^90^Sr activity concentrations, which were found in the water of Tributary 3 (T-3), reached 76000 Bq/kg and 130 Bq/kg, respectively.

#### 3.1.4 Water quality assessment of Karabulak Creek

To assess the quality of the surface water, The WCCI HM was applied, considering the correction for the hazard class [33]. The values derived from the WCCI HM are listed in Table 5 for each hazard class. The intervention levels exceeded those for ^3^H and ^90^Sr.

**Table 5:** Water contamination rate at Karabulak Creek.

Sampling point	WCCI HM class 1	WCCI HM class 2	WCCI HM class 3	Excess of IL for Sr-90, IL = 4.9 Bq/kg	Excess of IL for H-3, IL = 7600 Bq/kg
**Tributary 1**					
**T-1**	2.8 (Be and U)	2.1 (Al, Mo, Li)	1.3 (Fe)	3.3	5.8
**Tributary 2**					
**T-2**	3.9 (Be)	1.0 (Al)	1.5 (Fe, Mn)		
**Tributary 3**					
**T-3**	9.6 (Be and U)	-	-	27	10
**Tributary 4**					
**T-4**		1.0 (Li)	-	3.7	2.9
**T-5**	1.5 (Be)	-	-	5.3	3.3
**T-6**	2.5 (Be)	1.4 (Cd, Pb)	-		2.4
**Bed**					
**T-7**	-	1.2 (Al)	1.6 (Mn)		1.1
**T-8**	2.0 (Be)	-	-		2.5
**T-9**	-	-	-		2.5
**T-10**	2.3 (U)	4.3 (Mo, Li, Cd)	-		
**T-11**	2.6 (U)	4.7 (Li)	12 (Fe, Mn)		
**T-12**	-	2.3 (Li)	-		
**T-13**	1.6 (U)	1.7 (Li)	1.1 (Mn)		

Note:

Green: • regulatory clean waters

Orange: • heavily contaminated waters

Yellow: • moderately contaminated waters

Red: • extremely contaminated waters

In the waters of Karabulak Creek, elevated concentrations of beryllium and uranium, related to elements of hazard class 1, were detected. According to the gradation quoted in Table 1, points T-5 (Tributary 4), T-8, and T-13 (Bed) relate to the lowest contamination level of heavy metals of hazard class 1 –regulatory clean. Based on the WCCI HM of hazard class 1 (Table 1), The Karabulak Creek waters relate to the moderate level of water contamination for T-1 (Tributary 1), T-2 (Tributary 2), T-6 (Tributary 4), T-10, and T-11 (Bed). Based on the WCCI HM of hazard class 1, a high level of water contamination was recorded at point T-3 (Tributary 3), which contained high concentrations of beryllium and uranium.

A moderate level of water contamination for the group of heavy metals in hazard class 2 was revealed for T-1 (Tributary 1), T-10, T-11, T-12, and T-13 (Bed). The following points are among the regulatory clean water classes: T-2 (Tributary 2), T-4 and T-6 (Tributary 4), and T-7 and T-13 (Bed). According to the WCCI HM of hazard class 3, there is an extremely high level of water contamination in the Karabulak Creek for T-11 (Bed), which contains high concentrations of ferrum and manganese. The lowest levels of water contamination by heavy metals of hazard class 3 (regulatory clean) included points T-1 (Tributary 1), T-2 (Tributary 2), T-7, and T-13 (Bed).

Excess MPC for heavy metals was detected at all water sampling points of interest in Karabulak Creek, except at pointt-9 (Bed). For comparison, Table 4 shows The excess intervention levels for radionuclides, such as ^90^Sr and ^3^H. Elevated activity concentrations of these radionuclides, compared with The intervention level, were detected at most Tributary points of Karabulak Creek, other than Tributary 2 (T-2). AT The main bed section survey points, excess ^3^H was recorded only at T-7, T-8, and T-9. Concurrently, the most contaminated point was T-3, at which the excess IL for ^90^Sr and ^3^H were 27 and 10, respectively. The same point was The most contaminated for elements of hazard class 1 (beryllium and uranium). Point T-3 corresponds to one of the Karabulak heads and is located near Tunnel 511 (Fig 1), exhibiting external water seepage.

### 3.2 Discussion

Research undertaken in 2010 and 2011 [4] revealed high concentrations of beryllium, uranium, and molybdenum in the water of Karabulak Creek. Our data indicate an extremely non-uniform elemental composition of surface waters in Karabulak Creek, with elevated concentrations of a wide range of elements, including lithium, beryllium, ferrum, manganese, molybdenum, cadmium, and uranium.

**Fig 3:**
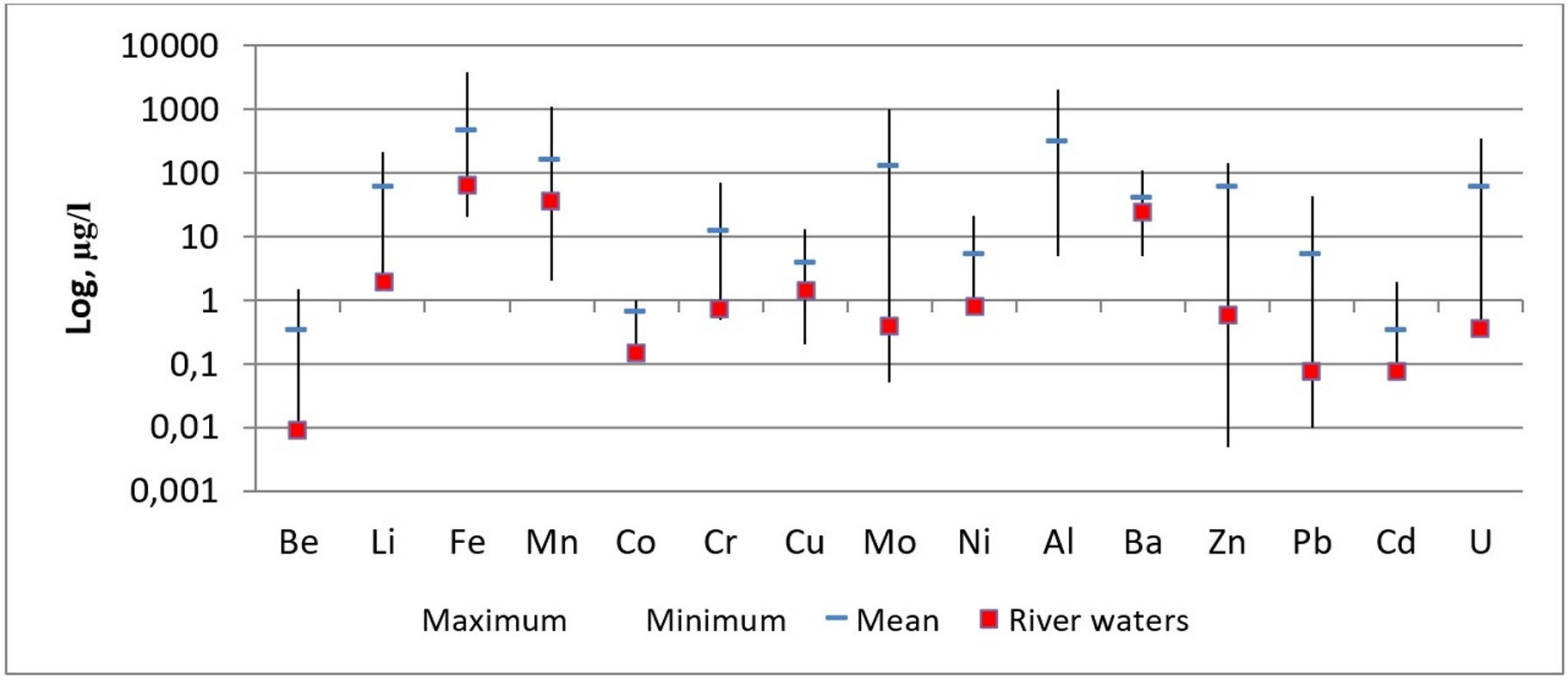
Spread interval and average content of chemical elements in Karabulak Creek compared to the average content in the river water [36].

To obtain information on the geochemical differences between waters in Karabulak Creek and the average content in river waters worldwide [36], a comparison graph was constructed (Table 1 in S1 Appendix, Fig 3).

The concentrations of the studied elements exceeded the mean values for global river waters: for uranium and molybdenum, hundreds of times; and for zinc, lead, beryllium, lithium, and chrome, dozens of times. Of special note is the uranium content, which is both a radioactive element and a chemical Toxicant that refers to hazard class 1 with the average concentration being 59 μg/L. Tests using fissile materials were conducted at the STS, where the charges contained both ^239^Pu and ^235^U [2, 37, 38]. Water samples containing high uranium concentrations were tested for uranium isotope composition (Table 6). According to our findings, the origin of uranium isotopic composition in the water of Karabulak Creek was natural (mass fraction of ^235^U = 0.72%).

**Table 6:** Uranium isotopic composition (^235^U and ^238^U).

Sampling point	^235^U (μg/L)	^238^U (μg/L)	^235^U (%)
T-1	0.57±0.03	78±4	0.72±0.03
T-3	2.52±0.13	347±17	0.73±0.02
T-10	1.01±0.05	139±7	0.72±0.03

An elevated content of uranium in the water and its natural origin were noted when studying Uzynbulak Creek at the «Degelen» site [8] and Tunnel waters (in 504, 506, and 511) of the same site [3]. Reportedly, the elevated content of this element is caused by numerous granitoid masses and fine uranium ore occurrences localised around the creek. Overall, the study area is referred to as the uranium province: in the north and east, the STS Territory borders the Kainar uranium-bearing region and the Symeitau uranium ore subzone [9].

**Fig 4:**
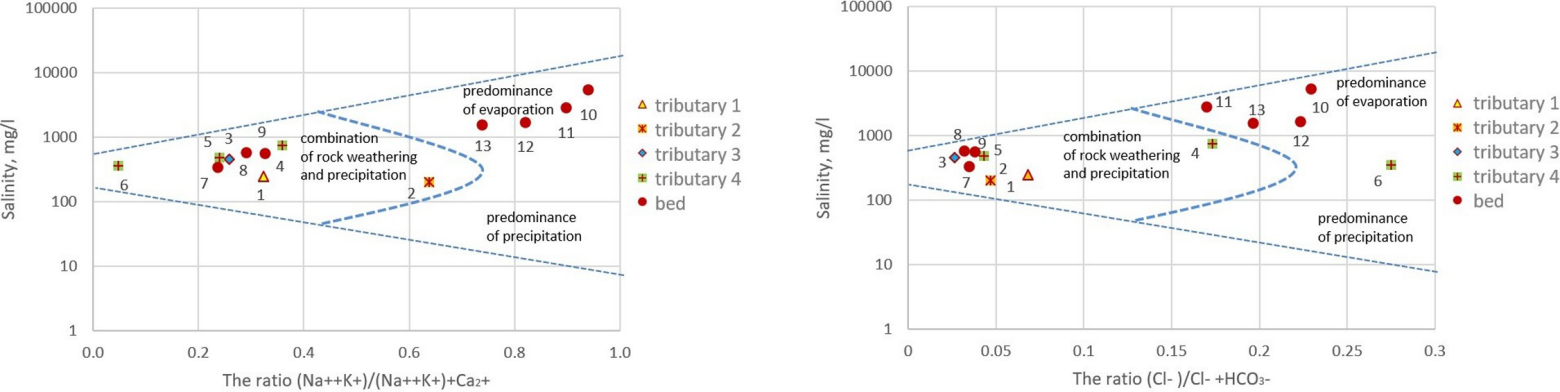
Gibbs diagram for the factors indicating hydrogeochemical processes in water of Karabulak Creek: a) dependency of (Na^+^+K^+^)/(Na^+^+K^+^+Ca^2+^) on TDS; b) dependency of Cl^–^/(Cl^–^+HCO3–) on TDS. Sampling points are marked with dots.

Gibbs diagram [39] (Fig 4) highlights the water-forming mechanisms that depend on three different factors: evaporative concentration, rock weathering, and water dilution by precipitation.

The main mechanisms affecting the chemical composition of the Karabulak Tributaries, based on the concentration ratios of major water ions, are rock weathering and precipitation. Evaporative concentration had a dominant effect on Karabulak water in the main bed (p.10, p.11, p.12, and p.13), as shown by the high water salinity (Table 2).

The formation of such a chemical composition is due to the fact that the water stream is in the granite massif and intersects the fluorite deposit «Karadzhal», which fosters the enrichment of surface waters with elements. It is worth noting that hydrophilic rocks in The study region were leached under conditions of elevated fracturing and the formation of a large number of crushing zones due to deformation at the Degelen mountain range during nuclear Tests [14]. One important factor is the mechanism of evaporative concentration and continental salinisation specific to surface water bodies in arid zones.

While addressing the contamination of Karabulak water with artificial radionuclides, a non-uniform contamination pattern is noteworthy, where the maximum activity concentrations of ^3^H and ^90^Sr were recorded in Tributary 3 (Table 4). The Third Tributary was also the most contaminated water stream flow of Karabulak Creek, the valley of which hosts tunnel 511, with a peak concentration of ^90^Sr in the water reaching 400 Bq/kg and containing ^137^Cs up to 700 Bq/kg [21]. Of the three creeks surveyed at the «Degelen» site (Karabulak, Uzynbulak and Baitles), continually elevated ^3^H values in the water (54000 to 57000 Bq/kg) were determined in the monitoring site (BMS-1) located at Tributary 3 of Karabulak Creek [40].

**Fig 5:**
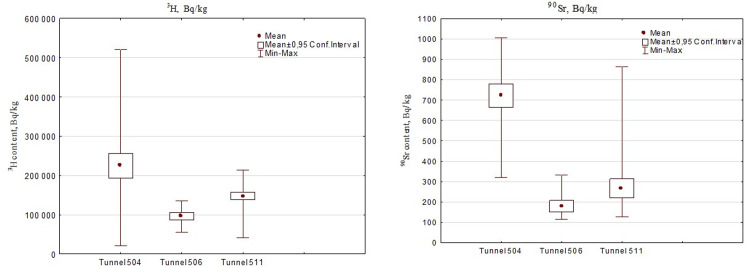
Long-term monitoring data (2001–2007) on the content of ^3^H and ^90^Sr in waters of Tunnels with a steady water seepage.

High concentrations of ^90^Sr and ^3^H in water samples of Karabulak Creek are caused by existing Tunnels exhibiting a steady water seepage where the creek is flowing (No. 504, 506, and 511). The monitoring data for these Tunnels from 2001–2007 are depicted in Table 2 in S1 Appendix, Fig 5.

High levels of ^3^H and ^90^Sr were observed in the water of Tunnels 504, 506, and 511. The average content of ^90^Sr was 36 to 147 times the intervention level (IL = 4.9 Bq/kg) and for ^3^H it was 13 to 30 Times (IL = 7600 Bq/kg). Peak concentrations of ^90^Sr and ^3^H were observed in the water of Tunnel No. 504, reaching 720 and 225000 Bq/kg, respectively.

Tunnels 504 and 506 were located close to sampling points 4, 5, and 6 (Tributary 4). High concentrations of ^90^Sr at sampling points T-1 (Tributary 1) and T-3 (Tributary 3), and ^3^H at T-1 (Tributary 1), T-7, T-8, and T-9 (Bed) were recorded at Tunnel 511.

## 4. Conclusions

This study found contamination of Karabulak Creek water both by artificial radionuclides such as ^3^H and ^90^Sr and chemical elements of hazard class 1, 2, and 3. Elevated activity concentrations of these radionuclides, compared to the intervention level, were detected mainly at sampling points in the Tributaries of Karabulak Creek. In the main bed section, the excess was recorded only for ^3^H at T-7, T-8, and T-9. The cause of high concentrations of ^90^Sr and ^3^H in water samples of Karabulak Creek is attributed to the presence of Tunnels with a water seepage where the creek is flowing.

At most survey water sampling points at Karabulak Creek, MPCs were exceeded for the following elements: Be (1.5–7.5 Times), Li (1–7 Times), Fe (1.3–13 Times), Mn (1.1–11 Times), Mo (1.1–4 Times), Cd (1.1–2 Times), and U (1.6–12 Times). However, the origin of the isotopic composition of uranium in the water of Karabulak Creek is natural (mass fraction of ^235^U = 0.72%). According to the WCCI HM, in most cases, a moderate level of water contamination of Karabulak Creek was observed for hazard class 1 (Be and U) and hazard class 2 (Al, Mo, Li, and Cd). According to the WCCI HM of hazard class 3, a high level of water contamination of Karabulak Creek was revealed for T-3 (Be and U) and extremely high contamination levels for T-11 (Fe and Mn).

Due to exceeded regulatory values for health-related, toxicological, and radiation hazard criteria, the waters of Karabulak Creek are unusable for economic purposes, in accordance with the Health Standards established by the Republic of Kazakhstan. Therefore, as a recommendation, it would be advisable for the local population to avoid watering animals at the most «contaminated» points (T-3, T-1), especially as they contain radionuclides (^90^Sr, ^3^H) and elements of hazard class 1 (Be, U).

The ecological situation around Karabulak Creek at the «Degelen» site, where underground nuclear blasts were conducted, is unique and attributed to a combined influence of man-made radiation and natural origin factors, in particular the geochemical features of the region. In addition, one of the consequences caused by underground nuclear blasts at the «Degelen» site is an increased migration of natural and artificial radionuclides and chemical elements in general due to a change in hydrological and geochemical conditions.

## Supporting information

S1 Appendix (DOCX)
