# Supplementary material for: Assessment of contamination of natural waters with radionuclides and heavy metals the case of Karabulak creek at the Semipalatinsk Test Site
Source: PLoS One. 2025 Feb 6;20(2):e0310833. doi: 10.1371/journal.pone.0310833 (PMC11801561; doi:10.1371/journal.pone.0310833)
Supplement: S1 Appendix — (DOCX) [file pone.0310833.s001.docx]

**Table 1. The values used to build graphs Fig 3. Spread interval and the average content of chemical elements in the Karabulak creek compared to the average content in the river water**

|  | Be | Li | Fe | Mn | Co | Cr | Cu | Mo | Ni | Al | Ba | Zn | Pb | Cd | U |
| --- | --- | --- | --- | --- | --- | --- | --- | --- | --- | --- | --- | --- | --- | --- | --- |
| Maximum | 1,5 | 210 | 3900 | 1100 | 1 | 69 | 13 | 1000 | 21 | 2000 | 110 | 140 | 43 | 1,9 | 350 |
| Minimum | 0,02 | 11 | 20 | 2 | 0,39 | 0,5 | 0,2 | 0,05 | 1 | 5 | 5 | 0,005 | 0,01 | 0,1 | 0,7 |
| Mean | 0,32 | 57 | 455 | 152 | 0,63 | 11,6 | 3,9 | 124 | 5,2 | 312 | 38 | 55 | 5 | 0,34 | 59 |
| River waters | 0,009 | 1,84 | 66 | 34 | 0,15 | 0,7 | 1,48 | 0,42 | 0,8 | - | 23 | 0,6 | 0,08 | 0,08 | 0,37 |

**Table 2.** **The values used to build graphs Fig 5. Long-term monitoring data (2001-2007) on the content of ^3^H and ^90^Sr in tunnels of steady water seepage**

| № | 504 tunnel, Bk/kg (water) | | 506 tunnel, Bk/kg (water) | | 511 tunnel, Bk/kg  (water) | |
| --- | --- | --- | --- | --- | --- | --- |
|  | ^90^Sr (n=31) | ^3^H (n=34) | ^90^Sr (n=26) | ^3^H (n=30) | ^90^Sr (n=32) | ^3^H (n=78) |
| 1 | 637 | 261000 | 123 | 109000 | 266 | 164900 |
| 2 | 750 | 252000 | 131 | 127000 | 210 | 213000 |
| 3 | 657 | 190000 | 128 | 120000 | 251 | 182000 |
| 4 | 631 | 199000 | 115 | 128000 | 249 | 129000 |
| 5 | 753 | 400000 | 126 | 109000 | 307 | 186800 |
| 6 | 686 | 152000 | 129 | 118000 | 296 | 42000 |
| 7 | 549 | 309000 | 126 | 136000 | 299 | 212000 |
| 8 | 762 | 299000 | 141 | 114810 | 224 | 168600 |
| 9 | 873 | 297000 | 194 | 107000 | 281 | 165000 |
| 10 | 606 | 319140 | 333 | 119000 | 216 | 150000 |
| 11 | 319 | 521000 | 333 | 107000 | 127 | 155300 |
| 12 | 672 | 185000 | 333 | 125000 | 148 | 159200 |
| 13 | 496 | 239000 | 206 | 120000 | 249 | 163000 |
| 14 | 829 | 213000 | 206 | 107000 | 211 | 159000 |
| 15 | 936 | 269000 | 206 | 55000 | 279 | 190000 |
| 16 | 936 | 262000 | 242 | 111000 | 260 | 183000 |
| 17 | 936 | 287000 | 242 | 62000 | 171 | 187000 |
| 18 | 692 | 308000 | 117 | 59000 | 247 | 176000 |
| 19 | 692 | 169000 | 242 | 56000 | 189 | 179000 |
| 20 | 692 | 221000 | 117 | 82000 | 863 | 194000 |
| 21 | 677 | 130000 | 114 | 57000 | 273 | 186000 |
| 22 | 677 | 148000 | 114 | 80000 | 267 | 175000 |
| 23 | 731 | 139000 | 114 | 85000 | 267 | 190000 |
| 24 | 677 | 259000 | 168 | 95000 | 267 | 175000 |
| 25 | 731 | 138000 | 168 | 88000 | 193 | 178000 |
| 26 | 586 | 240000 | 185 | 87000 | 193 | 176000 |
| 27 | 482 | 248000 | - | 63000 | 193 | 187000 |
| 28 | 1006 | 271000 | - | 88000 | 399 | 187000 |
| 29 | 1006 | 157000 | - | 90000 | 399 | 187000 |
| 30 | 870 | 20000 | - | 86051 | 170 | 178000 |
| 31 | 790 | 199504 | - | - | 399 | 182000 |
| 32 | - | 175298 | - | - | 170 | 175000 |
| 33 | - | 200000 | - | - | - | 178000 |
| 34 | - | 130000 | - | - | - | 183000 |
| 35 | - | - | - | - | - | 178000 |
| 36 | - | - | - | - | - | 179000 |
| 37 | - | - | - | - | - | 181000 |
| 38 | - | - | - | - | - | 179000 |
| 39 | - | - | - | - | - | 179000 |
| 40 | - | - | - | - | - | 179000 |
| 41 | - | - | - | - | - | 181000 |
| 42 | - | - | - | - | - | 180000 |
| 43 | - | - | - | - | - | 181000 |
| 44 | - | - | - | - | - | 168760 |
| 45 | - | - | - | - | - | 151920 |
| 46 | - | - | - | - | - | 174000 |
| 47 | - | - | - | - | - | 150900 |
| 48 | - | - | - | - | - | 163000 |
| 49 | - | - | - | - | - | 181000 |
| 50 | - | - | - | - | - | 139000 |
| 51 | - | - | - | - | - | 135000 |
| 52 | - | - | - | - | - | 138000 |
| 53 | - | - | - | - | - | 135000 |
| 54 | - | - | - | - | - | 145000 |
| 55 | - | - | - | - | - | 106000 |
| 56 | - | - | - | - | - | 101000 |
| 57 | - | - | - | - | - | 64000 |
| 58 | - | - | - | - | - | 67000 |
| 59 | - | - | - | - | - | 65000 |
| 60 | - | - | - | - | - | 121000 |
| 61 | - | - | - | - | - | 66000 |
| 62 | - | - | - | - | - | 108000 |
| 63 | - | - | - | - | - | 121000 |
| 64 | - | - | - | - | - | 119000 |
| 65 | - | - | - | - | - | 123000 |
| 66 | - | - | - | - | - | 115000 |
| 67 | - | - | - | - | - | 116000 |
| 68 | - | - | - | - | - | 113000 |
| 69 | - | - | - | - | - | 71000 |
| 70 | - | - | - | - | - | 118000 |
| 71 | - | - | - | - | - | 117000 |
| 72 | - | - | - | - | - | 94166 |
| 73 | - | - | - | - | - | 88858 |
| 74 | - | - | - | - | - | 101184 |
| 75 | - | - | - | - | - | 89814 |
| 76 | - | - | - | - | - | 100000 |
| 77 | - | - | - | - | - | 80000 |
| 78 | - | - | - | - | - | 62000 |
| **Mean** | **721** | **229616** | **179** | **96362** | **267** | **147710** |
| **Min** | **319** | **20000** | **114** | **55000** | **127** | **42000** |
| **Maх** | **1006** | **521000** | **333** | **136000** | **863** | **213000** |
